# Intra-Arterial Injection of Thrombin as Rescue Therapy of Vessel Perforation during Mechanical Thrombectomy for Acute Ischemic Stroke

**DOI:** 10.3390/brainsci12060760

**Published:** 2022-06-10

**Authors:** Tingyu Yi, Wenhuo Chen, Yanmin Wu, Zhinan Pan, Xiaohui Lin, Dinglai Lin, Rongcheng Chen, Xiufeng Zheng

**Affiliations:** Zhangzhou Affiliated Hospital of Fujian Medical University, Zhangzhou 363000, China; siyuyufen@163.com (T.Y.); minmindoc@163.com (Y.W.); m18050700089@163.com (Z.P.); linxh@foxmail.com (X.L.); lindinglai1@163.com (D.L.); crc337617@163.com (R.C.); zxf5860@163.com (X.Z.)

**Keywords:** acute ischemic stroke, vessel perforation, mechanical thrombectomy

## Abstract

Background: Vessel perforation during stent mechanical thrombectomy (MT) is a rare and disastrous complication. A routine rescue strategy includes balloon occlusion for tamponade, procedure suspension, and lowering or normalizing blood pressure. However, this complication is still associated with poor outcome and high mortality. Objective: We present our experience with intra-arterial injection of thrombin in the treatment of vessel perforation secondary to microcatheter/microwire perforation, which prevents further deterioration in clinical outcomes. Methods: Cases with intraprocedural vessel perforation during mechanical thrombectomy were included in the final analysis. Clinical data, procedural details, and radiographic and clinical outcomes were collected. Results: Four patients with intraprocedural vessel perforation were included. Intraprocedural perforations occurred at the distal middle cerebral artery in two cases: the A2 segment in one case and the internal carotid artery terminus in one case. The etiology of four cases was intracranial atherosclerotic stenosis (ICAS). The ruptured vessels were effectively occluded in all cases. Endovascular therapy was continued in three cases, and mTICI ≥ 2b recanalization was achieved in all cases. The culprit artery was kept patent on CTA for 72 h post-operation. No active bleeding was detected on follow-up CT post-operation. During the 90-day follow-up period, one patient died, modified Rankle Scare (mRS) 3 was observed in two patients, and mRS 4 was observed in one patient. Conclusions: The key benefit of this method is occluding the ruptured vessel without affecting the following MT. We propose that intra-arterial injection of prothrombin may be simple yet effective in managing vessel perforation complications during MT.

## 1. Introduction

Endovascular therapy is a standard and effective treatment for acute intracranial large artery occlusion (LAO) [1,2,3,4,5,6,7]. Complications of endovascular therapy include intracranial hemorrhage (ICH), vasospasm, air emboli, emboli to new territories, arterial dissection, and serious groin complications [8,9,10]. The mechanisms of ICH include direct endoluminal trauma and shear forces on the perforator vessels, which cause subarachnoid hemorrhage (SAH), ischemic and reperfusion injury, and parenchymal hematoma formation [11,12,13]. Vessel perforation during stent retriever thrombectomy is a rare and disastrous complication, and the rate is 0–4.0% [1,2,3,4,5,8,11,14,15,16,17,18]. When vessel perforation occurs, the routine rescue strategy includes balloon occlusion for tamponade, vessel sacrifice with endovascular coil or glue, procedure suspension, and lowering or normalizing blood pressure.

In this paper, we describe a method of selective microcatheter injection of thrombin to occlude vessel perforation secondary to microcatheters/microwires to prevent further neurologic deterioration. To the best of our knowledge, the aforementioned technique has not been reported to treat ruptured vessels in acute ischemic thrombectomy.

## 2. Materials and Methods

**Figure 1:**
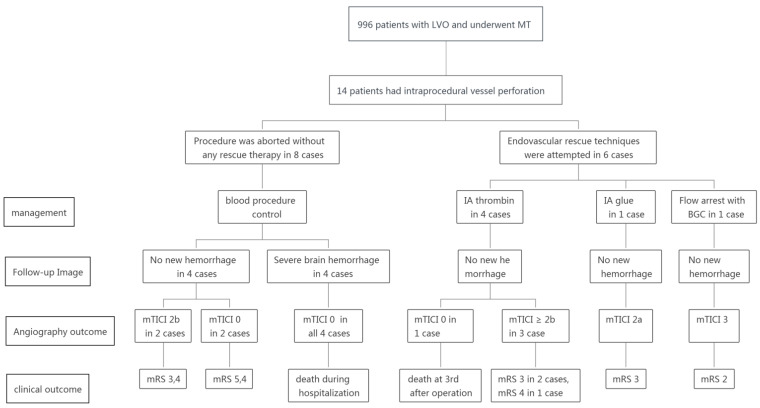
Diagram of management and outcome of patients with intraprocedural vessel perforation. LVO: large-vessel occlusion; MT: mechanical thrombectomy; IA: intraarterial; BGC: balloon-guided catheter; mRS: modified thrombolysis in cerebral infarction; NA: not applicable.

We reviewed our prospectively collected database of MT for AIS due to large-vessel occlusion from January 2015 to October 2019. This study was approved by our local ethics committee (the number is ID 2021 LWB251). In total, 996 consecutive patients with acute anterior large vessel occlusion underwent MT during this time in our institution. The endovascular technique was selected by the attending neuro-interventionalist based on the available therapies at the time of angiography. The strategies included mechanical retrievable stent thrombectomy, direct aspiration, angioplasty via a balloon or stent, or a combination of these approaches. Intraprocedural vessel perforation occurred in 14 patients, the procedure was aborted with no rescue endovascular treatment in 8 patients, extravasations ceased when the microwire was gently withdrawn from the perforator in 4 patients, and extravasations continued in the other 4 patients, all of whom later died (Figure 1).

Rescue endovascular treatment was adopted in six patients. One patient received rescue glue embolization with reperfusion therapy as aborted with modified thrombolysis in cerebral infarction (mTICI) 2A. The extravasations ceased after flow arrest with a balloon-guided catheter (BGC) in one patient, and reperfusion therapy continued to achieve mTICI 3 reperfusion. The other four patients received a selective microcatheter injection of thrombin. Technical details of each of the 14 thrombectomy cases, rescue approaches, and outcomes are described in Table 1 and Figure 1. The descriptions of the four patients who received selective microcatheter injection of thrombin are given as follows.

## 3. Cases 1 and 2: Middle Cerebral Artery (MCA) M1 Occlusion

**Figure 2:**
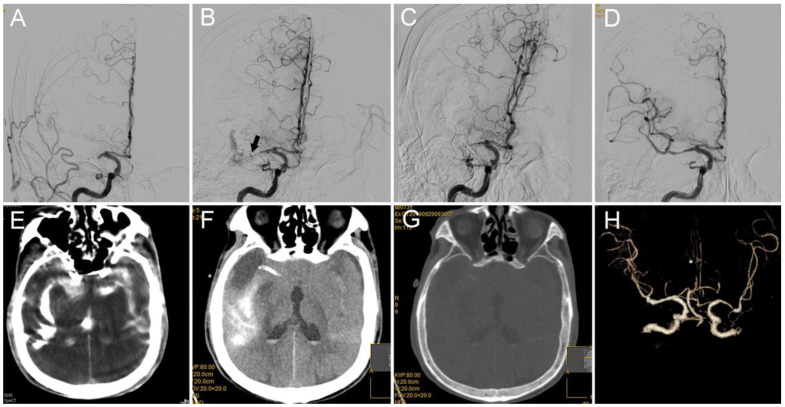
(**A**) DSA shows occlusion of the M1 segment of the right MCA. (**B**) A microcatheter is advanced into the M1/2 branch (the arrow indicates the tip of the microcatheter), where active contrast extravasation pointing to the location of vessel perforation can be observed (indicated with arrow). (**C**) After the injection of 400 U thrombin via the microcatheter and no following contrast extravasation, the contrast extravasation is stopped. (**D**) mTICI 3 reperfusion was achieved after the detachment of the stent retriever and administration of intravenous tirofiban. (**E**) Severe hyperdensity within the Sylvian fissure and ventricle was observed on C-arm CT performed during the procedure. (**F**,**G**) The hyperdense area was dismissed on follow-up CT and disappeared on dual-energy CT, which indicates that it was a contrast agent but not a blood product. (**H**) Patency of the culprit artery was observed on the follow-up CTA. MCA: middle cerebral artery; mTICI: modified thrombolysis in cerebral infarction; CTA: computed tomography angiography.

Case 1: In this case of acute stroke from a right MCA M1 occlusion, a 73-year-old male smoker with hypertension and hypertriglyceridemia presented with left limb weakness; the patient had an NIHSS score of 12 (Figure 2). Catheter angiography demonstrated persistent occlusion of the right MCA M1 segment (Figure 2A). During the procedure, the microcatheter “first-pass effect” [19] was observed, which indicated the MCA atherosclerosis-related occlusion. Then, an intravenous injection of tirofiban at a rate of 0.3 mg/h was administered after 0.5 mg intravenous bolus. After one thrombectomy pass with the 4–20 mm device (Medtronic), the right MCA M1 was recanalized with focused severe stenosis at the distal MCA, and angioplasty via a 2.0–15 mm balloon (Boston) was performed. Perforation at the M1/2 segment probably occurred due to the movement of the microwire. Angiography revealed severe contrast extravasation into the parenchyma after the microwire was retrieved back into the vessel (Figure 2B). The first step was to stop the administration of intravenous tirofiban. The second step was to advance the microcatheter into the place around the perforating site and subsequently retrieve the microwire. The third step was to inject 3 mL of thrombin (total dose of 400 u) into the microcatheter. Then, no contrast extravasation was detected during the observation time (Figure 2C). Ten minutes later, Solitaire 4–20 mm was re-unsheathed at the occlusion site; 20 min later, the intravenous injection of tirofiban was restarted at a rate of 0.25 mg/h; 1 h later, the stent was dethatched with mTICI 3 reperfusion achieved (Figure 2D). Hyperdensity within the Sylvian fissure and ventricle was detected on C-arm CT during the procedure (Figure 2E). No hemorrhage was detected by dual-energy CT performed after the procedure (Figure 2F,G). CTA performed 36 h post-procedure showed the culprit artery was patent (Figure 2H). Magnetic resonance angiography (MRA), performed 8 days post-procedure, showed right cerebral hemisphere hemorrhagic infarction with patent right MCA. The mRS was 2 at 3 months.

**Figure 3:**
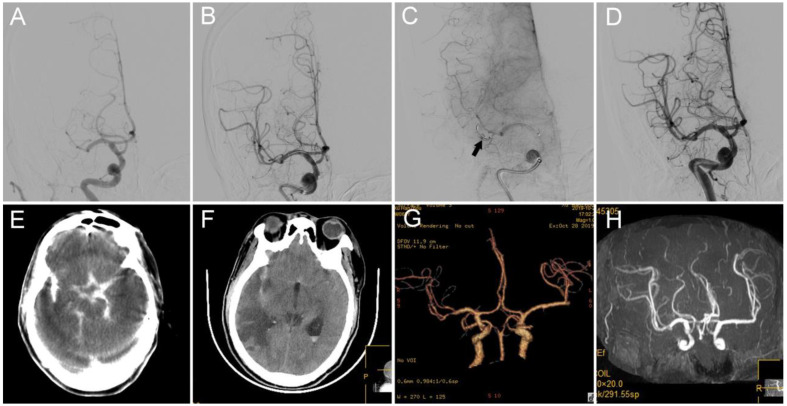
(**A**) DSA shows occlusion of the M1 segment of the right MCA. (**B**) The microcatheter advanced through the occlusion site, and a Solitaire 4–20 mm was unsheathed at the occlusion site. (**C**) Angiography showed contrast extravasation from the inferior trunk of the MCA (indicated by the black arrow). (**D**) No contrast extravasation after injection of 200 U thrombin via the microcatheter. mTICI 3 reperfusion was achieved when the microcatheter was re-advanced into the inferior trunk of the MCA over the microwire and retrieved back into the proximal segment of the MCA while the microwire remained in place. (**E**) Hyperdensity within the Sylvian fissure and ventricle was observed on C-arm CT performed during the procedure. (**F**,**G**) Hyperdense area dismissed on follow-up CT. (**G**,**H**) Patency of the culprit artery was observed on follow-up CTA and MRA. DSA: digital subtraction angiography; MCA, middle cerebral artery; mTICI: modified thrombolysis in cerebral infarction; CTA: computed tomography angiography.

Case 2: In this case of acute stroke from a right MCA M1 occlusion, a 76-year-old male patient with hypertension, diabetes mellitus, and atrial fibrillation presented with left limb weakness; the patient had a NIHSS score of 10 (Figure 3). Catheter angiography demonstrated persistent occlusion of the right MCA M1 segment (Figure 3A). The microcatheter “first-pass effect” was positive; then, an intravenous injection of tirofiban at a rate of 0.3 mg/h after 0.5 mg intravenous bolus was administered. After one thrombectomy pass with the 4–20 mm device (Medtronic), the occluded artery was opened (Figure 3B), but perforation at the M2 segment probably occurred due to retracement of the stent (Figure 3C). The first step was to stop the infusion of tirofiban. The second step was to advance the microcatheter over the microwire to the site near the perforator, retrieve the microwire, and inject 2 mL of thrombin (total dose of 200 u) into the microcrater. In the third step, the microwire was re-advanced into the distal segment and maintained in place. Then, the microcatheter was advanced into the distal site and immediately retrieved back to the M1 segment over the microwire. Angiography showed no contrast extravasation with patent of the culprit artery (Figure 3D). Five minutes later, the infusion of tirofiban was restarted at a rate of 0.25 mg/h. During the 30-min observation period, no new hemorrhage was detected, and mTICI grade 3 was achieved. Hyperdensity within the cisterna ambiens and sylvian cisterna was detected on C-arm CT during the procedure (Figure 3E). The hyperdense area was gradually dismissed on follow-up CT (Figure 3F), and CTA performed on the 8th day post-operation showed patent right MCA (Figure 3G). MRA performed on the 14th day post-operation showed right cerebral hemisphere hemorrhagic infarction with patent right MCA (Figure 3H). The mRS was 3 at 3 months.

## 4. Case 3: Internal Carotid Artery (ICA) Terminus Occlusion

**Figure 4:**
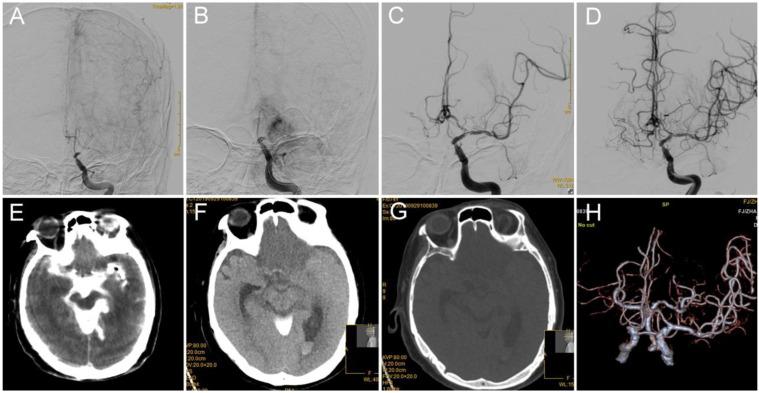
(**A**) DSA shows occlusion of the left ICA terminus. (**B**) Active contrast extravasation was observed when the microcatheter attempted to advance into the MCA, which indicates that vessel perforation occurred at the left carotid artery terminus. (**C**) After the injection of 200 µ thrombin via a microcatheter plus inflation of the balloon for 20 min, contrast extravasation was stopped, and the occluded left carotid artery terminus was opened. (**D**) mTICI 3 reperfusion was achieved 10 min after the administration of intravenous tirofiban. (**E**) Hyperdensity within the Sylvian fissure and cisterna ambiens was observed on C-arm CT performed during the procedure. (**F**,**G**) The hyperdense area was dismissed on follow-up CT and disappeared on dual-energy CT, which indicates that it was a contrast agent but not a blood product. (**H**) Patency of the culprit artery was observed on follow-up CTA. ICA: internal carotid artery; DSA: digital subtraction angiography; mTICI: modified thrombolysis in cerebral infarction; CTA: computed tomography angiography.

In this case of acute stroke from the left ICA terminus occlusion, a 74-year-old female patient with hypertension and diabetes mellitus presented with right limb weakness; the patient had a NIHSS score of 21 (Figure 4). Catheter angiography demonstrated persistent occlusion of the left ICA terminus (Figure 4A). Perforation at the ICA terminus probably occurred because the microwire and microcatheter were mistakenly advanced into the parenchyma (Figure 4B). The first step was to maintain the microwire in place, retrieve the microcatheter back to the perforated orifice, and subsequently inject 2 mL of thrombin (total dose of 200 u) into the microcatheter. The second step was to advance another microwire (Boston) using the microcatheter (Enchelon10, EV3) through the occlusion site, and angioplasty via 2.0–15 mm balloon (Boston) was performed. The third step was to retrieve the first microwire. Ten minutes after the inflation of the balloon, the microcatheter was gently retrieved. Twenty minutes later, the balloon was deflated, and no contrast extravasation was detected during the entire observation period (Figure 4C). Twenty-five minutes later, an intravenous injection of tirofiban was administered at a rate of 0.25 mg/h after 0.375 mg intravenous bolus. The anterograde blood flow through the lesion site improved, and mTICI 2b was achieved 15 min after the tirofiban administration. The bilateral anterior cerebral artery was supplied by the left ICA (Figure 4D). The systolic pressure was strictly controlled at 130 mmHg. Hyperdensity within the Sylvian fissure and cisterna ambiens was detected on C-arm CT during the procedure (Figure 4E). The hyperdense area was gradually dismissed on the follow-up CT, but a bilateral frontal lobe large infarction was detected on the follow-up CT (Figure 4F,G). CTA performed 48-h post-procedure showed that the culprit artery was patent (Figure 4H). The patient was discharged on the fourth-day post-operation, and the mRS score was 5 at discharge.

## 5. Case 4: Anterior Cerebral Artery (ACA) A2 Segment

**Figure 5:**
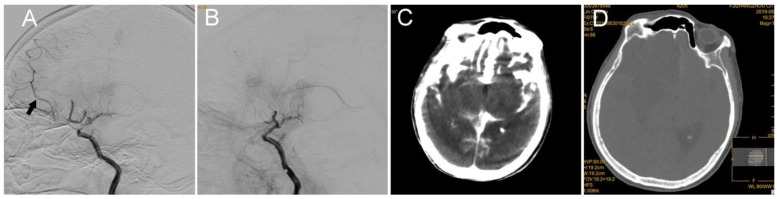
(**A**) Vessel perforation occurred when attempting to advance the microcatheter into A3; the microcatheter was in the parenchyma (the black arrow indicates the tip of the microcatheter). DSA shows the occlusion of the A2 segment of the left anterior cerebral artery. (**B**) This ruptured vessel was immediately sealed with 2 mL of thrombin (total dose was 300 u). (**C**) C-arm CT showed hyperdensity in the cisterna ambiens and sylvian cisterna. (**D**) Post-procedure dual-energy CT showed bilateral frontal lobe infarction without new hemorrhage or brain edema.

In this case of acute stroke from the left ACA A2 segment occlusion, a 76-year-old female patient with hypertension presented with left limb weakness; the patient had an NIHSS score of 6 (Figure 5). Catheter angiography demonstrated persistent occlusion of the left ACA A2 segment. Perforation at A1/A2 probably occurred because the microwire and microcatheter were mistakenly advanced into the parenchyma (Figure 5A). Microcatheter angiography revealed contrast extravasation into the parenchyma, which confirmed artery perforation at A2. First, a microwire was placed back in the parenchyma, and the microcatheter was retrieved back to the site around the perforator. Second, the microwire was retrieved, 2 mL of thrombin (total dose of 300 u) was injected into the microcatheter, and the ruptured vessel was immediately sealed (Figure 5B). Endovascular therapy was terminated. A hyperdense area within the cisterna ambiens and sylvian cisterna was detected on C-arm CT during the procedure (Figure 5C). Bilateral frontal lobe infarction and brain edema occurred, but no new hemorrhage was detected on dual-energy CT post-operation (Figure 5D). The patient was discharged on the second day and died on the third day after the operation.

## 6. Discussion

Vessel perforation is a rare and serious complication of endovascular therapy, and it requires urgent management because patients are at high risk of hemorrhagic stroke and further deterioration. Moshayedi proposed an approach to control cerebral vessel perforation and summarized it as follows. When intraprocedural vessel perforation occurs, the first step is to reverse the anticoagulants with reversal agents, reduce the blood pressure, and gently withdraw the offending hardware. If extravasations continue, intracranial balloon inflation is used to tamponade the perforation. If extravasations continue, super-selective catheterization of the perforation site and perforation sealing with Onyx glue injection is performed, or vessel sacrifice with endovascular coil or glue should be performed if these interventions do not work [20,21]. Flow arrest with a balloon-guided catheter is another method [14].

Two key factors should be considered in treating patients with vessel perforation: effective sealing of the vessel perforator and patency of the occluded artery. Studies have shown glue embolization of vessel perforation with preserved vessel patency during mechanical thrombectomy for acute ischemic stroke [20,21]. However, glue is a cohesive liquid, the microcatheter may adhere to the vessel, which makes it difficult to withdraw, and the microcatheter may be occluded by glue. Additionally, the parent artery of the perforating site may be occluded due to glue liquid reflux; thus, glue injection requires a selected technical note and should be performed by an experienced neurointerventionist.

Thrombin is one of the most potent platelet activators and a direct promoter of fibrin clot formation. Thrombin is most often used as a topical hemostatic agent. Three types of topical thrombin are available: human plasma-derived (h-thrombin), bovine plasma-derived (b-thrombin), and recombinant (r-thrombin) [22]. The hemostasis rate within 10 min of thrombin application was 88% [22]. Although all three forms of topical thrombin carry a specific warning of “do not inject” due to concern for intravascular thrombus propagation, they are used off-label for percutaneous injections to cause thrombosis primarily of iatrogenic femoral pseudoaneurysms [23,24]. The catheter-delivered endovascular use of thrombin to cause thrombosis of deep artery pseudoaneurysms was also reported [22,25,26]. Catheterization-related arterial perforation can be sealed by the catheter-delivered endovascular use of thrombin [27,28,29]. To the best of our knowledge, our case series is the first study to show the effectiveness and safety of intra-arterial injection of thrombin in sealing cerebral arterial perforation caused by microwires/microcatheters. This technique offers certain advantages. First, its effectiveness in hemostasis to arterial perforation is highly important. Second, the simplicity of this technique indicates that it can be widely used to treat arterial perforation. Third, compared with glue embolization, we do not fear the complication of the viscous liquid. Fourth, this technique precludes the need to hold the concomitant use of antithrombotic or antiplatelet agents [27].

Selected problems may remain a concern, e.g., the injection dose, injection concentration, and injection techniques. The injection dose is 250–5000 U [22,25,27,28,30], and the dose in our cases was 200–400 U. The injection concentration was 100–1000 U/mL [22,28,30], and the injection concentration was 100 U/mL in our cases. Certain skills are required in injecting thrombin. Thrombin was mixed with the patient’s blood to make a “thrombin-blood patch’’ (TBP), and the TBP was selectively injected through a microcatheter to the target site to occlude flow through the perforation track. This approach was believed to minimize the risk of embolization to the distal vessels [27]. When the infusion of thrombin was complete, a notably small (0.5 mL) bolus of air was intentionally injected through the microcatheter to further diminish the retrograde movement of thrombin or anterograde blood flow into the diagonal branch. The microcatheter was flushed with 2 mL of normal saline to clear any residual air and/or thrombin from the lumen [28]. The microcatheter was flushed with 2 mL of normal saline in our cases.

The clinical outcome may be more likely to be positive in patients with vessel preformation if at least partial recanalization (TICI 2a or higher) is achieved and hemorrhage is controlled [14]. Successful reperfusion was achieved in three of our four cases, mRS grade 3 was achieved in two patients, and the clinical outcome of one patient was poor due to the strictly controlled blood pressure and multiple cerebral artery stenoses. This case also hints that the blood pressure cannot be controlled too low in a case with a poor cerebral artery.

Vessel perforation is a rare complication of stent retriever thrombectomy, and the rate is approximately 1.6% in five recent endovascular trials [14]. The vessel perforation rate in our study was 1.4%, which is consistent with a previous study.

This study is limited by the small number of patients. Additionally, the optimal volume, dose, and technique of administration of endovascular thrombin are unclear. However, due to its effectiveness, simplicity, and ease of mastery, the catheter-directed endovascular application of thrombin is feasible rescue therapy in treating vessel perforation during mechanical thrombectomy.

## Figures and Tables

**Table 1:** Summary of technical details of thrombectomy, rescue approaches, and outcomes in patients with intraprocedural vessel perforation.

pt	Occlusion Site TOAST	IV rtPA before; IV Heparin during Thrombectomy	General Anaesthesia Yes/No	Approach to Thrombectomy	Stent Retriever No. of Passes	Location of Perforation	Suspected Cause of Perforation	Rescue Endovascular Treatment	Postprocedure CT	Outcomes
1	R-M1P ICAS	IV heparin	No	Stent retriever + Balloon angioplasty	1	RM1D	Perforation with microwire/microcatheter when traversing lesion site	IA thrombin	No new hemorrhage	mTICI 3, mRS 4 at 3 months
2	L-A3 ICAS	IV heparin	No	NA	NA	L-A2	Perforation with microwire/microcatheter when traversing clot	IA thrombin	No new hemorrhage	mTICI 0; death at 3rd days after operation
3	L-ICA terminus ICAS	IV heparin	No	Balloon angioplasty	NA	L-ICA terminus	Perforation with microwire/microcatheter when traversing lesion site	IA thrombin	No new hemorrhage	mTICI 3, mRS 3 at discharge
4	R-M1D ICAS	IV heparin	No	Stent retriever	Solitaire 4 × 20 1 pass	R M1D	Resistance withdrawing stent retriever	IA thrombin	No new hemorrhage	mTICI 2b, mRS 3 at 3 months
5	L-ICA terminus CE	IV heparin	No	BGC + stent retriever	Solitaire 6 × 30 1 pass	LM1P	Perforation with microwire/microcatheter when traversing clot	Flow arrest with BGC	No new hemorrhage	mTICI 3, mRS 2 at 3 months
6	L-M1D CE	IV heparin	No	Stent retriever	Solitaire 4 × 20 4 passes	L M3	Perforation with microcatheter injection to confirm location	IA glue	No new hemorrhage	mTICI 2a, mRS 3 at 3 months
7	R M2 CE	IV heparin	No	Stent retriever	Solitaire 4 × 20 1 pass	RM3	Perforation with microwire when traversing clot	Procedure aborted, blood pressure control	No new hemorrhage	mTICI 2b, mRS 3 at 3 months
8	L-M2 CE	IV heparin	No	NA	NA	L M3	Perforation with microwire when traversing clot	Procedure aborted, blood pressure control	No new hemorrhage	mTICI 0, mRS 5 at discharge
9	L-ICA terminus ICAS	IV heparin	No	NA	NA	L-ICA terminus	Perforation with microwire when traversing lesion site	Procedure aborted, blood pressure control	No new hemorrhage	mTICI 0, mRS 4 at 3 months
10	L-M2 CE	IV heparin	Yes	NA	NA	L M3	Perforation with microwire when traversing clot	Procedure aborted, blood pressure control	No new hemorrhage	mTICI 2b, mRS 4 at 3 months
11	L-Carotid T * CE	IV heparin	No	NA	NA	L M1P	Perforation with microwire when traversing clot	Procedure aborted, blood pressure control	Severe brain hemorrhage	mTICI 0; death in hospital
12	L-Carotid L ** CE	IV heparin	Yes	NA	NA	L-ICA terminus	Perforation with wire when advancing guiding catheter	Procedure aborted, blood pressure control	Severe brain hemorrhage	mTICI 0; death in hospital
13	R-Carotid-L CE	IV heparin + IV rt-PA	Yes	NA	NA	R M1P	Perforation with microwire when traversing clot	Procedure aborted, blood pressure control	Severe brain hemorrhage	mTICI 0; death in hospital
14	L-M1P CE	IV heparin	Yes	NA	NA	L anterior choroidal artery	Perforation with microcatheter went into anterior choroidal artery	Procedure aborted, blood pressure control	Severe brain hemorrhage	mTICI 0; death in hospital

Pt indicates patient; Toast indicates Trial of Org 10172 in Acute Stroke Treatment; IV, intravenous; No, number; R, right; L, left; M1P, proximal segment of middle cerebral artery; ICAS, intracranial atherosclerotic stenosis; mTICI, modified Thrombolysis in Cerebral Infarction; mRS, modified Rankin Scale; A3, A3 segment of anterior cerebral artery; A2, A2 segment of anterior cerebral artery; ICA, internal carotid artery; NA, not available; M1D, distal segment of middle cerebral artery; CE, cardiac embolism; BGC, balloon guiding catheter; M2, M2 segment of middle cerebral artery; M3, M3 segment of middle cerebral artery. * indicates terminal ICA plus Middle cerebral artery and anterior cerebral artery, ** indicates terminal ICA plus middle cerebral artery.

## Data Availability

Not available.
